# 1-(2-Hy­droxy-5-meth­oxy­phen­yl)-3-methyl­but-2-en-1-one^1^

**DOI:** 10.1107/S1600536812042158

**Published:** 2012-10-13

**Authors:** Catherine Thomas Alexander, David Vargas, Frank R. Fronczek, Steven F. Watkins

**Affiliations:** a Department of Chemistry, Louisiana State University, Baton Rouge LA 70803-1804 USA

## Abstract

The title compound, C_12_H_14_O_3_, is a natural product derived from the medium-sized hawthorn *Crataegus persimilis* (’*prunifolia*’). The mean plane of the butene moiety is twisted by 13.27 (7)° with respect to the that of the dioxobenzaldehyde moiety. There is an intra­molecular hydrogen bond between the hydroxyl group and the carbonyl O atom.

## Related literature

For isolation from plant material, see: Castro *et al.* (1989 ▶). For the synthesis, see: Camps *et al.* (1985 ▶). For photolysis to form 4-chromanones, see: Primo *et al.* (1982 ▶). For a related structure, see: Zeller *et al.* (2010 ▶).
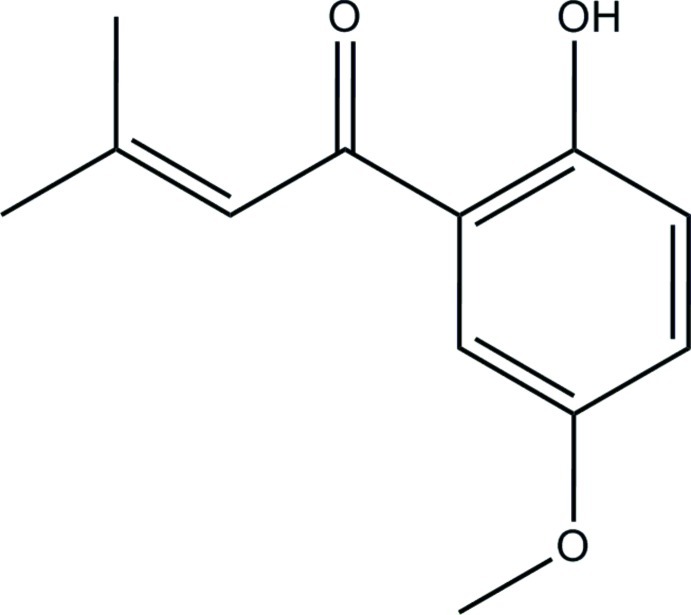


## Experimental

### 

#### Crystal data

C_12_H_14_O_3_
*M* *_r_* = 206.23
Monoclinic, 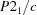

*a* = 14.027 (3) Å
*b* = 5.816 (1) Å
*c* = 12.829 (3) Å
β = 91.409 (8)°
*V* = 1046.3 (4) Å^3^
*Z* = 4
Mo *K*α radiation
μ = 0.09 mm^−1^
*T* = 100 K
0.45 × 0.37 × 0.23 mm

#### Data collection

Nonius KappaCCD diffractometer
Absorption correction: multi-scan (*HKL* *SCALEPACK*; Otwinowski & Minor 1997 ▶) *T*_min_ = 0.959, *T*_max_ = 0.979
6425 measured reflections
3784 independent reflections
3179 reflections with *I* > 2σ(*I*)
*R*_int_ = 0.018

#### Refinement

*R*[*F* ^2^ > 2σ(*F* ^2^)] = 0.043
*wR*(*F* ^2^) = 0.127
*S* = 1.04
3784 reflections
143 parameters
H atoms treated by a mixture of independent and constrained refinement
Δρ_max_ = 0.43 e Å^−3^
Δρ_min_ = −0.22 e Å^−3^

### 

Data collection: *COLLECT* (Nonius, 2000 ▶); cell refinement: *DENZO* and *SCALEPACK* (Otwinowski & Minor, 1997 ▶); data reduction: *DENZO* and *SCALEPACK*; program(s) used to solve structure: *SIR2002* (Burla *et al.*, 2003 ▶); program(s) used to refine structure: *SHELXL97* (Sheldrick, 2008 ▶); molecular graphics: *ORTEP-3 for Windows* (Farrugia, 1997 ▶); software used to prepare material for publication: *WinGX* publication routines (Farrugia, 1999 ▶).

## Supplementary Material

Crystal structure: contains datablock(s) global, I. DOI: 10.1107/S1600536812042158/im2405sup1.cif

Structure factors: contains datablock(s) I. DOI: 10.1107/S1600536812042158/im2405Isup2.hkl

Additional supplementary materials:  crystallographic information; 3D view; checkCIF report

## Figures and Tables

**Table 1:** Hydrogen-bond geometry (Å, °)

*D*—H⋯*A*	*D*—H	H⋯*A*	*D*⋯*A*	*D*—H⋯*A*
O2—H2*A*⋯O3	0.87 (2)	1.74 (2)	2.523 (1)	149 (2)

**Fig. 1.**
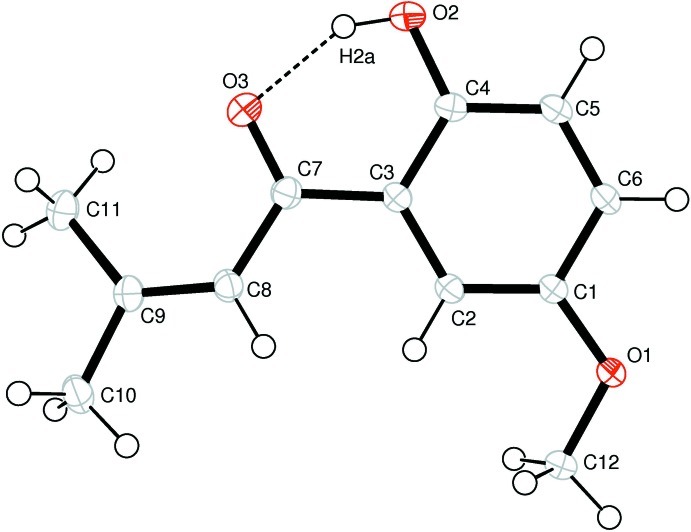
Molecular structure of (I) with displacement ellipsoids at the 50% probability level.

## supplementary crystallographic information

### Comment

The structure of title compound I can be described in terms of four planar moieties as defined by their constituent non-hydrogen atoms. The phenyl ring and three atoms bonded to it define the main molecular plane, with mean deviation of the defining atoms of δ_r.m.s._ = 0.0145 (6) Å. With respect to this molecular plane, the mean plane of the carbonyl group (four atoms, δ_r.m.s._ = 0.0044 (4) Å) and the plane of the methoxy group (three atoms) have dihedral angles of 2.50 (6)° and 4.33 (6)° respectively, while the mean plane of the butene moiety (four atoms, δ_r.m.s._ = 0.0018 (4) Å) has dihedral angle 13.27 (7)°.

### Experimental

Compound I was isolated as a natural product (Castro *et al.*, 1989). It has also been synthesized (Camps *et al.*, 1985). Suitable crystals were formed by very slow evaporation of a hexane solution over a period of three years.

### Refinement

The positional and isotropic displacement parameters of hydroxyl atom H2A were refined independently. All other H atoms were placed in calculated positions, guided by difference maps, and refined as riding. Torsional parameters for the three methyl groups were refined, with C—H = 0.98 Å and *U*_iso_(H) = 1.5*U*_eq_(C), while H atoms attached to *sp*^2^ C atoms have C—H = 0.95 Å and *U*_iso_(H) = 1.2*U*_eq_(C).

### Crystal data

**Table:**

C_12_H_14_O_3_	*F*(000) = 440
*M**_r_* = 206.23	*D*_x_ = 1.309 Mg m^−^^3^
Monoclinic, *P*2_1_/*c*	Mo *K*α radiation, λ = 0.71073 Å
Hall symbol: -P 2ybc	Cell parameters from 3442 reflections
*a* = 14.027 (3) Å	θ = 2.5–32.6°
*b* = 5.816 (1) Å	µ = 0.09 mm^−^^1^
*c* = 12.829 (3) Å	*T* = 100 K
β = 91.409 (8)°	Fragment, yellow
*V* = 1046.3 (4) Å^3^	0.45 × 0.37 × 0.23 mm
*Z* = 4	

### Data collection

**Table:**

Nonius KappaCCD diffractometer	3784 independent reflections
Radiation source: sealed tube	3179 reflections with *I* > 2σ(*I*)
Horizonally mounted graphite crystal monochromator	*R*_int_ = 0.018
Detector resolution: 9 pixels mm^-1^	θ_max_ = 32.6°, θ_min_ = 3.2°
φ and ω scans	*h* = −21→21
Absorption correction: multi-scan (*HKL**SCALEPACK*; Otwinowski & Minor 1997)	*k* = −8→7
*T*_min_ = 0.959, *T*_max_ = 0.979	*l* = −19→19
6425 measured reflections	

### Refinement

**Table:**

Refinement on *F*^2^	Primary atom site location: structure-invariant direct methods
Least-squares matrix: full	Secondary atom site location: difference Fourier map
*R*[*F*^2^ > 2σ(*F*^2^)] = 0.043	Hydrogen site location: inferred from neighbouring sites
*wR*(*F*^2^) = 0.127	H atoms treated by a mixture of independent and constrained refinement
*S* = 1.04	*w* = 1/[σ^2^(*F*_o_^2^) + (0.0692*P*)^2^ + 0.2297*P*] where *P* = (*F*_o_^2^ + 2*F*_c_^2^)/3
3784 reflections	(Δ/σ)_max_ = 0.001
143 parameters	Δρ_max_ = 0.43 e Å^−^^3^
0 restraints	Δρ_min_ = −0.22 e Å^−^^3^
0 constraints	

### Special details

**Table:**

Geometry. All e.s.d.'s (except the e.s.d. in the dihedral angle between two l.s. planes) are estimated using the full covariance matrix. The cell e.s.d.'s are taken into account individually in the estimation of e.s.d.'s in distances, angles and torsion angles; correlations between e.s.d.'s in cell parameters are only used when they are defined by crystal symmetry. An approximate (isotropic) treatment of cell e.s.d.'s is used for estimating e.s.d.'s involving l.s. planes.

### Fractional atomic coordinates and isotropic or equivalent isotropic displacement parameters (Å^2^)

**Table:**

	*x*	*y*	*z*	*U*_iso_*/*U*_eq_	
C1	0.38220 (6)	1.03873 (14)	0.88580 (6)	0.01713 (16)	
C2	0.31710 (6)	0.89827 (14)	0.93398 (6)	0.01652 (15)	
H2	0.3003	0.9281	1.0040	0.020*	
C3	0.27530 (6)	0.71019 (14)	0.87973 (6)	0.01641 (15)	
C4	0.30238 (6)	0.66752 (15)	0.77617 (6)	0.01796 (16)	
C5	0.36779 (6)	0.81313 (15)	0.72851 (6)	0.01992 (17)	
H5	0.3853	0.7853	0.6586	0.024*	
C6	0.40689 (6)	0.99610 (16)	0.78231 (6)	0.01999 (17)	
H6	0.4509	1.0944	0.7491	0.024*	
C7	0.20366 (6)	0.55840 (14)	0.92711 (7)	0.01901 (16)	
C8	0.17084 (6)	0.60621 (15)	1.03279 (7)	0.01893 (16)	
H8	0.1897	0.7485	1.0632	0.023*	
C9	0.11620 (6)	0.46622 (15)	1.09083 (7)	0.01933 (16)	
C10	0.08404 (7)	0.54666 (17)	1.19537 (7)	0.02467 (19)	
H10A	0.1069	0.7037	1.2079	0.037*	
H10B	0.1100	0.4446	1.2498	0.037*	
H10C	0.0142	0.5442	1.1967	0.037*	
C11	0.08255 (7)	0.23100 (16)	1.05970 (8)	0.02593 (19)	
H11A	0.0197	0.2424	1.0251	0.039*	
H11B	0.0781	0.1342	1.1219	0.039*	
H11C	0.1279	0.1625	1.0117	0.039*	
C12	0.40983 (6)	1.26466 (15)	1.03865 (7)	0.02072 (17)	
H12A	0.3425	1.3041	1.0465	0.031*	
H12B	0.4497	1.3925	1.0637	0.031*	
H12C	0.4249	1.1263	1.0794	0.031*	
O1	0.42786 (5)	1.22263 (11)	0.93148 (5)	0.02247 (15)	
O2	0.26717 (5)	0.49087 (12)	0.71825 (5)	0.02388 (15)	
H2A	0.2287 (12)	0.416 (3)	0.7580 (14)	0.052 (5)*	
O3	0.17042 (5)	0.39337 (13)	0.87547 (6)	0.02755 (16)	

### Atomic displacement parameters (Å^2^)

**Table:**

	*U*^11^	*U*^22^	*U*^33^	*U*^12^	*U*^13^	*U*^23^
C1	0.0188 (3)	0.0184 (3)	0.0143 (3)	−0.0008 (3)	0.0011 (3)	0.0000 (3)
C2	0.0168 (3)	0.0186 (3)	0.0142 (3)	0.0004 (3)	0.0009 (3)	0.0004 (3)
C3	0.0166 (3)	0.0175 (3)	0.0152 (3)	0.0007 (3)	0.0007 (3)	0.0006 (3)
C4	0.0195 (3)	0.0188 (3)	0.0156 (3)	0.0018 (3)	−0.0008 (3)	−0.0011 (3)
C5	0.0230 (4)	0.0234 (4)	0.0134 (3)	−0.0002 (3)	0.0018 (3)	−0.0006 (3)
C6	0.0226 (4)	0.0235 (4)	0.0140 (3)	−0.0026 (3)	0.0031 (3)	0.0010 (3)
C7	0.0181 (3)	0.0192 (4)	0.0198 (4)	−0.0002 (3)	0.0004 (3)	0.0002 (3)
C8	0.0179 (3)	0.0191 (3)	0.0199 (4)	−0.0006 (3)	0.0027 (3)	0.0002 (3)
C9	0.0157 (3)	0.0199 (4)	0.0224 (4)	0.0013 (3)	0.0007 (3)	0.0035 (3)
C10	0.0241 (4)	0.0267 (4)	0.0236 (4)	−0.0015 (3)	0.0071 (3)	0.0029 (3)
C11	0.0265 (4)	0.0208 (4)	0.0305 (5)	−0.0040 (3)	0.0023 (4)	0.0039 (3)
C12	0.0243 (4)	0.0223 (4)	0.0157 (3)	−0.0029 (3)	0.0026 (3)	−0.0027 (3)
O1	0.0286 (3)	0.0238 (3)	0.0153 (3)	−0.0094 (2)	0.0052 (2)	−0.0030 (2)
O2	0.0301 (3)	0.0225 (3)	0.0190 (3)	−0.0042 (3)	0.0017 (3)	−0.0051 (2)
O3	0.0317 (4)	0.0260 (3)	0.0251 (3)	−0.0101 (3)	0.0034 (3)	−0.0049 (3)

### Geometric parameters (Å, º)

**Table:**

C1—O1	1.3708 (10)	C8—H8	0.95
C1—C2	1.3824 (11)	C9—C11	1.4982 (13)
C1—C6	1.4023 (12)	C9—C10	1.5003 (13)
C2—C3	1.4159 (11)	C10—H10A	0.98
C2—H2	0.95	C10—H10B	0.98
C3—C4	1.4127 (12)	C10—H10C	0.98
C3—C7	1.4795 (12)	C11—H11A	0.98
C4—O2	1.3540 (10)	C11—H11B	0.98
C4—C5	1.4002 (12)	C11—H11C	0.98
C5—C6	1.3751 (12)	C12—O1	1.4251 (11)
C5—H5	0.95	C12—H12A	0.98
C6—H6	0.95	C12—H12B	0.98
C7—O3	1.2499 (11)	C12—H12C	0.98
C7—C8	1.4690 (12)	O2—H2A	0.870 (18)
C8—C9	1.3538 (12)		
			
O1—C1—C2	125.22 (7)	C8—C9—C11	125.55 (8)
O1—C1—C6	114.77 (7)	C8—C9—C10	119.40 (8)
C2—C1—C6	120.00 (8)	C11—C9—C10	115.05 (8)
C1—C2—C3	120.46 (7)	C9—C10—H10A	109.5
C1—C2—H2	119.8	C9—C10—H10B	109.5
C3—C2—H2	119.8	H10A—C10—H10B	109.5
C4—C3—C2	118.71 (7)	C9—C10—H10C	109.5
C4—C3—C7	118.86 (7)	H10A—C10—H10C	109.5
C2—C3—C7	122.43 (7)	H10B—C10—H10C	109.5
O2—C4—C5	116.96 (8)	C9—C11—H11A	109.5
O2—C4—C3	123.13 (8)	C9—C11—H11B	109.5
C5—C4—C3	119.91 (8)	H11A—C11—H11B	109.5
C6—C5—C4	120.43 (8)	C9—C11—H11C	109.5
C6—C5—H5	119.8	H11A—C11—H11C	109.5
C4—C5—H5	119.8	H11B—C11—H11C	109.5
C5—C6—C1	120.48 (8)	O1—C12—H12A	109.5
C5—C6—H6	119.8	O1—C12—H12B	109.5
C1—C6—H6	119.8	H12A—C12—H12B	109.5
O3—C7—C8	120.88 (8)	O1—C12—H12C	109.5
O3—C7—C3	119.26 (8)	H12A—C12—H12C	109.5
C8—C7—C3	119.85 (7)	H12B—C12—H12C	109.5
C9—C8—C7	126.04 (8)	C1—O1—C12	117.01 (7)
C9—C8—H8	117	C4—O2—H2A	106.4 (12)
C7—C8—H8	117		
			
O1—C1—C2—C3	−178.95 (8)	C2—C1—C6—C5	−0.92 (13)
C6—C1—C2—C3	0.33 (13)	C4—C3—C7—O3	1.59 (12)
C1—C2—C3—C4	0.82 (12)	C2—C3—C7—O3	−179.06 (8)
C1—C2—C3—C7	−178.54 (8)	C4—C3—C7—C8	−176.96 (7)
C2—C3—C4—O2	179.35 (7)	C2—C3—C7—C8	2.39 (12)
C7—C3—C4—O2	−1.28 (12)	O3—C7—C8—C9	11.11 (14)
C2—C3—C4—C5	−1.39 (12)	C3—C7—C8—C9	−170.37 (8)
C7—C3—C4—C5	177.99 (8)	C7—C8—C9—C11	2.89 (14)
O2—C4—C5—C6	−179.87 (8)	C7—C8—C9—C10	−176.50 (8)
C3—C4—C5—C6	0.82 (13)	C2—C1—O1—C12	3.14 (12)
C4—C5—C6—C1	0.34 (13)	C6—C1—O1—C12	−176.18 (8)
O1—C1—C6—C5	178.43 (8)		

### Hydrogen-bond geometry (Å, º)

**Table:**

*D*—H···*A*	*D*—H	H···*A*	*D*···*A*	*D*—H···*A*
O2—H2*A*···O3	0.87 (2)	1.74 (2)	2.523 (1)	149 (2)
